# Identification of MYC and STAT3 for early diagnosis based on the long noncoding RNA-mRNA network and bioinformatics in colorectal cancer

**DOI:** 10.3389/fimmu.2024.1497919

**Published:** 2025-01-03

**Authors:** Kunhou Yao, Hao Fan, Tiancheng Yang, Can Yang, Guibin Wang, Xingwang Li, Xin-Ying Ji, Qun Wang, Shaojiang Lv, Shihao Guo

**Affiliations:** ^1^ Department of General Surgery, Huaihe Hospital of Henan University, Henan University, Kaifeng, Henan, China; ^2^ School of Basic Medicine, Henan University, Kaifeng, Henan, China; ^3^ Department of General Surgery, Huaxian County People’s Hospital, Huaxian, Henan, China; ^4^ Department of Colorectal Surgery, First Affiliated Hospital of Zhengzhou University, Zhengzhou, Henan, China

**Keywords:** colorectal cancer, lncRNA, ceRNA, hub genes, biomarkers

## Abstract

**Background:**

Colorectal cancer (CRC) ranks among the top three cancers globally in both incidence and mortality, posing a significant public health challenge. Most CRC cases are diagnosed at intermediate to advanced stages, and reliable biomarkers for early detection are lacking. Long non-coding RNAs (lncRNAs) have been implicated in various cancers, including CRC, playing key roles in tumor development, progression, and prognosis.

**Methods:**

A comprehensive search of the PubMed database was conducted to identify relevant studies on the early diagnosis of CRC. Bioinformatics analysis was performed to explore lncRNA-mRNA networks, leading to the identification of five potential blood biomarkers. Expression analysis was carried out using the GEPIA and GEO online databases, focusing on MYC and STAT3. Differential expression between normal and CRC tissues was assessed, followed by Receiver Operating Characteristic (ROC) analysis to evaluate the diagnostic potential of these markers. Quantitative Real-Time PCR (qRT-PCR) was performed to validate MYC and STAT3 expression levels, and findings were further confirmed using the Human Protein Atlas (HPA) database.

**Results:**

Database analysis revealed significant differential expression of MYC and STAT3 between normal and CRC tissues. ROC analysis demonstrated the diagnostic potential of these markers. qRT-PCR validation confirmed the differential expression patterns observed in the databases. Validation through the HPA database further supported these findings, confirming the potential of MYC and STAT3 as diagnostic biomarkers for CRC.

**Conclusion:**

Our results suggest that MYC and STAT3 are promising diagnostic biomarkers for CRC, offering new insights into its pathophysiology and potential for targeted therapies.

## 1 Introduction

CRC is a malignancy that arises in the colon or rectum, with the rectum being the most frequently affected site. The predominant histological type is adenocarcinoma, although squamous cell carcinoma can also occur but is less common. CRC is more prevalent in middle-aged and elderly individuals, with a higher incidence in men compared to women. Currently, the main treatment modalities for CRC are surgery, endoscopic treatment, radiotherapy and chemotherapy with 5-fluorouracil, platinum, hydroxycamptothecin, vincristine, methotrexate, irinotecan, paclitaxel and/or cetuximab; however, there are defects and drawbacks in these modalities such as infections, damage to the liver and renal functions, dissemination of tumors, affecting the hematopoietic system, and chemoresistance, etc (1). As of today, the field of CRC treatment and management worldwide suffers from problems and drawbacks such as untimely diagnosis, inadequate screening, limitations in drug therapy, side effects of surgical procedures, low cure rate of metastatic CRC, and poor prognostic outcomes (2). Therefore there is a need for more in-depth studies such as the role of epigenetics in the inflammation-cancer transition, development of transcriptomic technologies to provide better diagnostic and prognostic utility and identification of disease-specific biomarkers (3, 4).

LncRNAs are a major class of non-coding RNAs that have received increasing attention in recent years, are involved in complex cancer networks, and have a large role in the diagnosis, treatment, and prognosis of CRC, and may have oncogenic or oncostatic functions (5–7). Through different modes of action such as proliferation, apoptosis, DNA repair, drug resistance, and metastasis, lncRNAs appear to be abnormally expressed and affect the radiosensitivity and survival of malignant tumor cells (8, 9). lncRNAs, significant pathogenic factors to epigenetic mechanisms, are engaged in colorectal carcinogenesis, metastasis, and progression, and are involved in the regulation of autophagy, allowing them to adjust the response of the cancer cells to chemotherapeutic modalities (10, 11). Abnormal expression of many miRNAs is also participated in CRC progression, and there is evidence that they have dual roles in oncogenesis and cancer inhibition, depending on the surrounded cellular environment (12).

The ceRNA network describes the complex post-transcriptional communication network of all transcribed RNA species, including lncRNAs, which can act as natural miRNA sponges to inhibit miRNA function by sharing miRNA response elements (MRE) (13).The ceRNA network is involved in many biological processes, including epithelial-mesenchymal transition (EMT), proliferation, apoptosis, metastasis, and chemotherapy resistance (14). So far, the specific ceRNA regulation mechanism of lncRNA-miRNA-mRNA in CRC remains unclear.

The MYC gene, which consists of three paralogenics, C-MYC, N-MYC and L-MYC (15), controls global gene expression and regulates cell proliferation, cell differentiation, cell cycle, metabolism and apoptosis (16).There is strong evidence that aberrant MYC expression is a driver of tumorigenesis and maintenance (17). Dysregulated gene expression is not only due to chromosomal translocations or copy number alterations involving MYC genes, but also because MYC is downstream of multiple oncogene signaling pathways. For example, dysregulated WNT signaling in colorectal tumors always results in high MYC levels. Thus, MYC expression above physiologically permissible thresholds can induce tumor development or strongly accelerate tumorigenesis in multiple tissues. The inhibition of MYC for cancer therapy is a very active area of preclinical research.

Signal transducer and activator of transcription 3 (STAT3) plays an important role in intracellular signaling (18). The lncRNAs in the ceRNA network that they regulate indirectly affect STAT3 expression through competitive binding with specific miRNAs (19). This regulatory mechanism has been reflected in a variety of disease models, especially in cancer, inflammatory response and stem cell differentiation. In breast cancer, the miR-17-5p/STAT3/H19 axis was found to regulate the expression of STAT3, thereby affecting the proliferation and metastasis of cancer cells. In addition, lncRNAs also interact with miRNAs as ceRNA in a variety of pathological models to regulate the expression of STAT3. For example, in the process of angiogenesis in colorectal cancer, lncRNAs affect STAT3 by interacting with miRNAs, thereby affecting the migration ability of cancer cells (20). Under the regulation of lncRNA, STAT3 shows significant biological effects in a variety of cell states and disease models (21). These findings not only deepen the understanding of ceRNA mechanisms, but also provide new targets for disease treatment.

In this paper, we studied the expression level of lncRNAs in colorectal cancer; discussed the relationship of related potential lncRNAs with miRNAs and mRNAs and constructed a ceRNA network. By studying the function and mechanism of action of lncRNAs in colorectal cancer, relevant genes were identified as biomarkers based on the ceRNA network. All the discussed studies offer strong potential for future diagnosis and treatment of colorectal cancer.

## 2 Materials and methods

### 2.1 Retrieval strategy

We searched PubMed for studies that serve as diagnostic, prognostic, or predictive biomarkers for CRC. Five search terms were used for the paper search with the following search terms: (“Long non-coding RNA”) AND (CRC OR colorectal cancer OR colorectal neoplasm OR colorectal tumor OR colitis-associated Neoplasms), (“Linc RNA”) AND (CRC OR colorectal cancer OR colorectal neoplasm OR colorectal tumor OR colitis-associated Neoplasms), (“Lnc RNA”) AND (CRC OR colorectal cancer OR colorectal neoplasm OR colorectal tumor OR colitis-associated Neoplasms), (“Long intergenic non-protein coding RNA”) AND (CRC OR colorectal cancer OR colorectal neoplasm OR colorectal tumor OR colitis-associated Neoplasms), (“Long non-protein-coding RNA”) AND (CRC OR colorectal cancer OR colorectal neoplasm OR colorectal tumor OR colitis-associated Neoplasms), and by using the five search styles, we obtained a large number of documents to ensure a complete search rate and screened the retrieved documents for elimination.

### 2.2 Eligibility criteria

The studies which are eligible should meet the following criteria (1): identification of CRC by the gold criteria, and(2) independent initial studies assessing the expression of lncRNAs in CRC tumor tissue as a means of evaluating diagnostic, treatment response, predictive, or prognostic biomarkers in the patient population. Exclusion criteria were (1) raw letter articles (2) written in non-English (3) non-human studies; (4) reviews, letters, comments, commentaries, or meta-analyses; (5)other diseases research, (6)non-colorectal cancer studies; (7) no studies of lncRNAs; and (8) simultaneous studies of multiple lncRNAs. All assessments were independently conducted to ensure accurate study inclusion.

### 2.3 Analysis of protein-protein interaction networks and hub genes

PPIs were constructed using STRING, which is a public online protein-protein interaction analysis tool. The screened differentially expressed genes were imported into the STRING database to estimate PPI, followed by generating visual network maps of the PPIs using Cytoscape software, and co-screening of pivotal genes using CytoHubba, CytoNCA and MCODE plug-ins in Cytoscape.

### 2.4 Gene expression analysis

Online differential gene expression analysis was performed in the GEPIA database. Meanwhile, we selected GSE39582 data from the GEO database and performed differential gene expression analysis using R (4.4.1). ROC analysis was then performed to determine the diagnostic value of selected centrally located genes in colorectal cancer based on the GSE10950 dataset of the GEO database. We used immunohistochemistry results from the HPA database to further verify gene expression levels.

### 2.5 qRT-PCR verification

(1) The system configuration is shown in the table below (**Table 1**):

**Table 1:** System configuration for qRT-PCR validation.

reagents	volume of use(μL)
SYBR qPCR Master Mix	10
Forward Primer	0.5
Reward Primer	0.5
DNase-Free ddH_2_O	8
cDNA	1
Total volume	20

(2) After mixing, add the PCR reaction plate, on the machine, the program is set as follows (**Table 2**):

**Table 2:** Process of PCR reaction.

Stage 1	Reps: 1	95°C	5 min
Stage 2	Reps: 40	95°C	10 sec
		8°C	30 sec
		72°C	30 sec
Stage 3	Reps: 1	72-95°C	0.5°C/sec

(3) Exporting data for analysis.

## 3 Results

### 3.1 Search result

**Figure 1:**
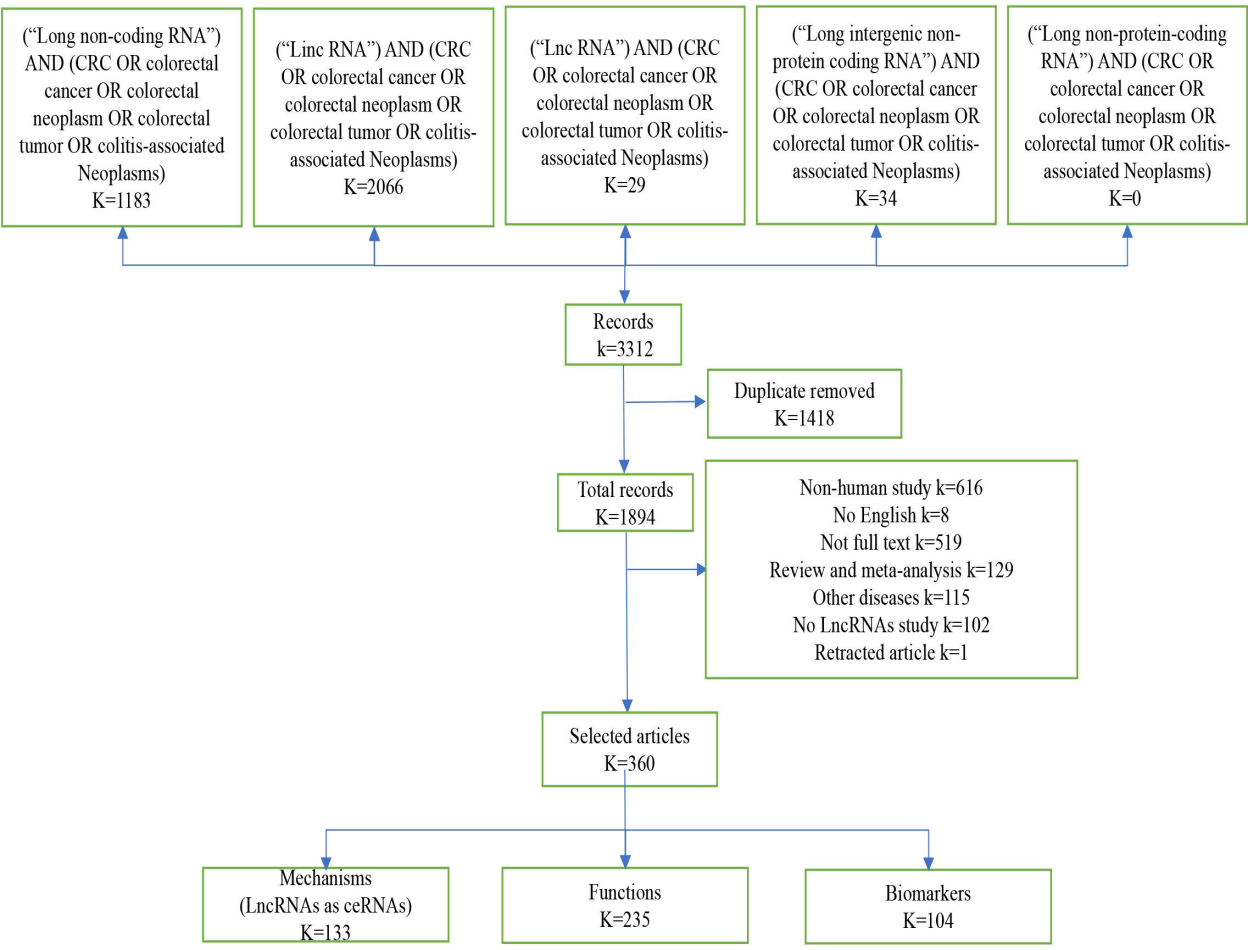
Screening method. Diagram “K” means the number of records, by screening the literature, we obtained 360 eligible publications on lncRNA and colorectal cancer in this article.

**Figure 1** shows the selection procedure for lncRNA identification, inclusion and exclusion criteria. We retrieved 3312 studies from the PubMed database, of which 1418 duplicates were excluded. Next, we based the inclusion and exclusion criteria, namely, articles in non-English language, articles whose full text could not be consulted or withdrawn, literature type as review or meta-analysis, research content as other diseases or non-human or no mention of lncRNA, etc. Next, we based the inclusion and exclusion criteria, namely, non-English language, full text not be consulted or withdrawn, type of literature as review or meta-analysis, studies on other diseases or non-human or no mention of lncRNA, etc. After reading the titles and abstracts, a total of 1634 records were excluded (**Figure 1**). A total of 360 publications were eventually included in this systematic review. Among these 360 papers, lncRNAs as competing endogenous RNAs (ceRNAs) were 133, 235 investigated the function of lncRNAs, 67 oncogenes, 168 tumor suppressors, and a total of 104 markers, 39 diagnostic biomarkers, 53 prognostic biomarkers, and 12 therapeutic predictive biomarkers (**Figure 1**).

### 3.2 ceRNA regulatory network

**Figure 2:**
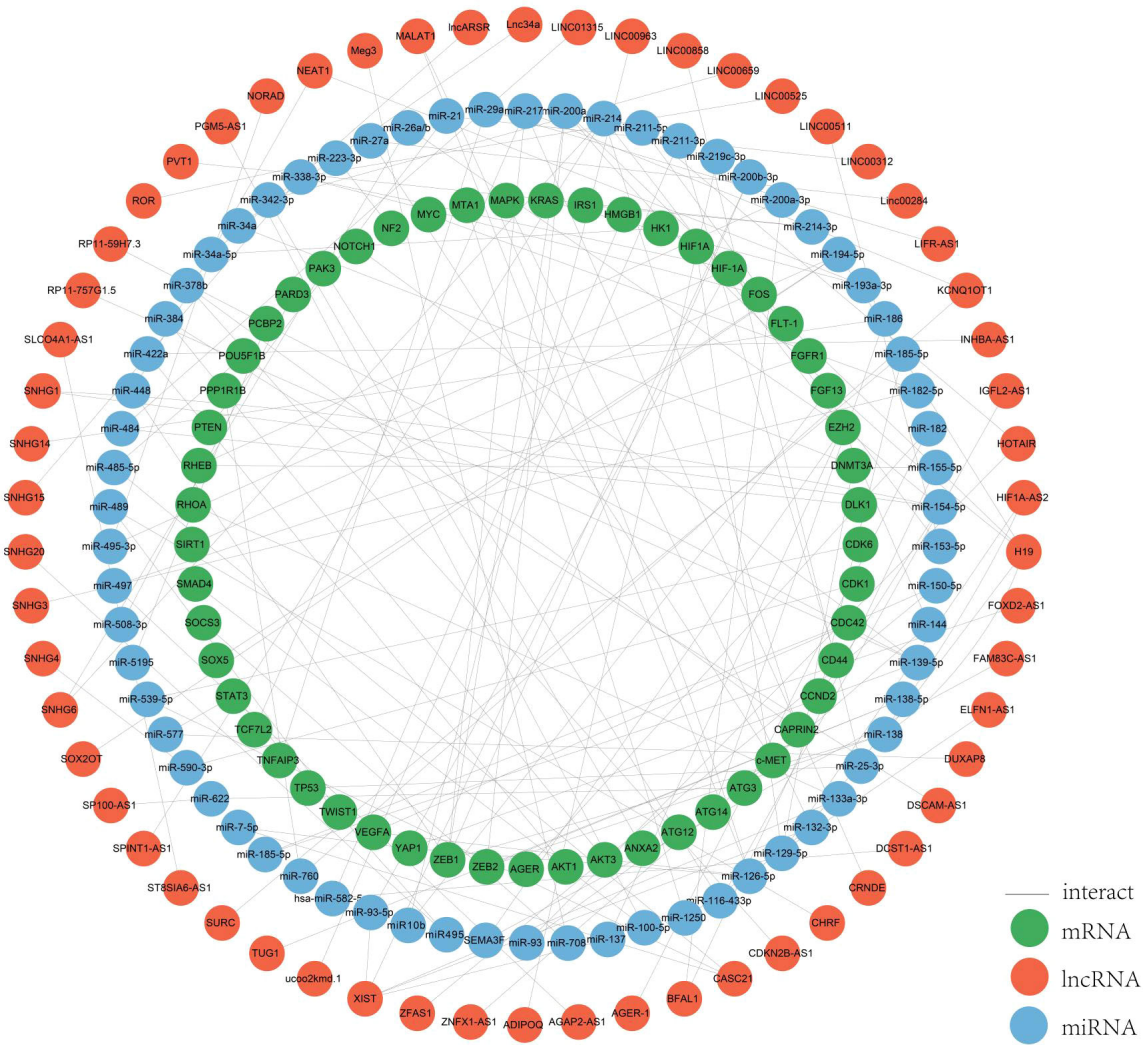
ceRNA network. We mapped this network through the relationships regulated by the ceRNA network by summarizing the relationships between the lncRNAs, miRNAs and mRNAs we screened for.

In colorectal cancer, lncRNAs play a key role in regulating the occurrence and development of cancer, and are involved in a variety of regulatory mechanisms, such as promoting the epigenetic silencing or activation of target genes in epigenetic aspects, participating in the development of colorectal cancer as pseudogenes, the lncRNA-protein axis is involved in colorectal cancer. LncRNAs can be used as structural components to regulate protein activity or change protein localization by binding to specific proteins containing nuclear transcription factors, and the lncRNA-miRNA-mRNA axis regulates CRC cell proliferation, metastasis and drug resistance, among others. LncRNAs can be used as structural components to regulate protein activity or alter protein localization by binding to specific proteins containing nuclear transcription factors; lncRNA-miRNA-mRNA axis regulates CRC cell proliferation, metastasis and drug resistance, etc. (22, 23). Among all mechanisms of CRC, the ceRNAs network has received more and more attention in recent years. In the ceRNA network, the intracellular regulation of miRNAs is mainly in the form of negative regulation to regulate gene expression levels, and there are various miRNA response elements, MRE, in the transcripts of coding genes, which can bind to the miRNAs and lead to degradation of the mRNAs or inhibition of their translation (24–26). When lncRNAs act as ceRNAs which are natural miRNA sponges that share the same MRE as the mRNA, the lncRNAs compete to occupy the commonly shared binding sequences of the miRNAs, thereby sequestering the miRNAs and altering the expression of their downstream target genes (27). The level of intracellular lncRNA expression directly affects the amount of miRNA that can be bound by the corresponding mRNA. Thus, lncRNAs indirectly regulate the expression level of mRNAs through the bridge of MRE, thus regulating cellular functions (26). We systematically counted the ceRNA network in the CRC, and more than 100 studies analyzed 76 lncRNAs as ceRNAs forming a ceRNA network with miRNAs and mRNAs (**Figure 2**). Among them, SNHG3 regulates CRC development by regulating proliferation, differentiation and apoptosis, CASC21 regulates CRC development by regulating cell growth, and XIST and NEAT regulate tumor histogenesis and migration by regulating cell migration. The results of the study are as follows.

SNHG3 is a non-coding RNA involved in cell proliferation, differentiation and apoptosis, and plays a role in gene transcription and translation, which binds to specific DNA sequences and regulates the expression of certain genes. The expression of SNHG3 is up-regulated in colorectal cancer tissues and cell lines, and its high level is associated with advanced colorectal cancer stage, positive lymph node metastasis and poor prognosis. Some researchers verified the role of CAFs-EVs containing SNHG3 *in vivo* by establishing a xenograft tumor model and found that SNHG3 promotes CRC cell proliferation by increasing HuR expression through the sponge miR-34b-5p and ultimately enhancing HOXC6 transcription (28, 29). Moreover, SNHG3 further induces CRC proliferation and invasion by interacting with miR-370-5p and thus participating in the regulation of EZH1 expression (28).

CASC21 promotes the growth of CRC cells as an oncogene and is overexpressed in CRC, which is associated with low survival. CASC21 regulates its target CDK6 by sponging on miR-539-5p, which may serve as a exotic target for CRC treatment and diagnosis. CASC21 regulates YAP1 and CDK6 expression by sponging miR-7-5p and miR-539-5p (30). Meanwhile, YAP1 expression is controlled by two other lncRNAs, MALAT1 and RP11-59H7.3, in combination with two different miRNAs (31, 32).

XIST is associated with tumor progression and its expression is upregulated in CRC, and its overexpression promotes migration and invasive potential (33). In response to miR-93-5p, XIST adjusts the expression of HIF-1A and promotes the EMT process, proliferation and migration and tumorigenesis of colorectal cancer, suggesting that XIST may serve as a diagnostic and therapeutically relevant marker (34). In addition, XIST binds to miR-137, and the enhancer of zeste homolog 2 (EZH2) is a target of miR-137 is repressed expression, which ultimately promotes tumor metastasis in colorectal cancer (33). XIST can be regarded as a prognostic indicator of CRC progression. XIST regulates the expression of four mRNAs, EZH2, RHOA, and EZB1, through sponging of other miRNAs (34, 35), XIST can be used as a prognostic indicator for CRC progression.

NEAT1 (nuclear parasite assembly transcript 1) promotes the proliferation and migration of CRC cells, and aberrant expression in CRC tissues and cells leads to a poor prognosis, and improves the proliferation and migration of CRC cells (36). Sponge acts on miR-448 to regulate EZB1 expression. Some researchers demonstrated that miR-196a-5p inhibited glial cell-derived neurotrophic factor (GDNF) expression in HCT116 cells using qPCR and western blot analysis. miR-196a-5p can bind to NEAT1’s predicted binding site, and thus NEAT1 exerts its oncogenic effects through the miR-196a-5p/GDNF axis in CRC (37–39).

### 3.3 Functions of lncRNAs in CRC

LncRNAs have emerged as key players in cancer, influencing various aspects of tumor cell behavior including metastasis, proliferation, apoptosis, drug resistance, and epithelial-mesenchymal transition (EMT) processes (40–42). In tumor cells, lncRNAs are frequently aberrantly expressed. Abnormally expressed lncRNAs can participate in numerous pathological events, notably contributing to the development and progression of cancer.

There are fourteen recognized characteristics of cancer: evasion of growth inhibitory factors, avoidance of immune destruction, induction of angiogenesis, immortalization of replication, continuous growth signaling, resistance to apoptosis, epigenetic alterations, activation of spread and metastasis, genetic changes and variations, inflammation induced by tumors, reversion of cellular specialization and transformation into a different cell type, abnormal epigenetic modifications, microbiota alterations, and changes in neural communication (43, 44).

**Figure 3:**
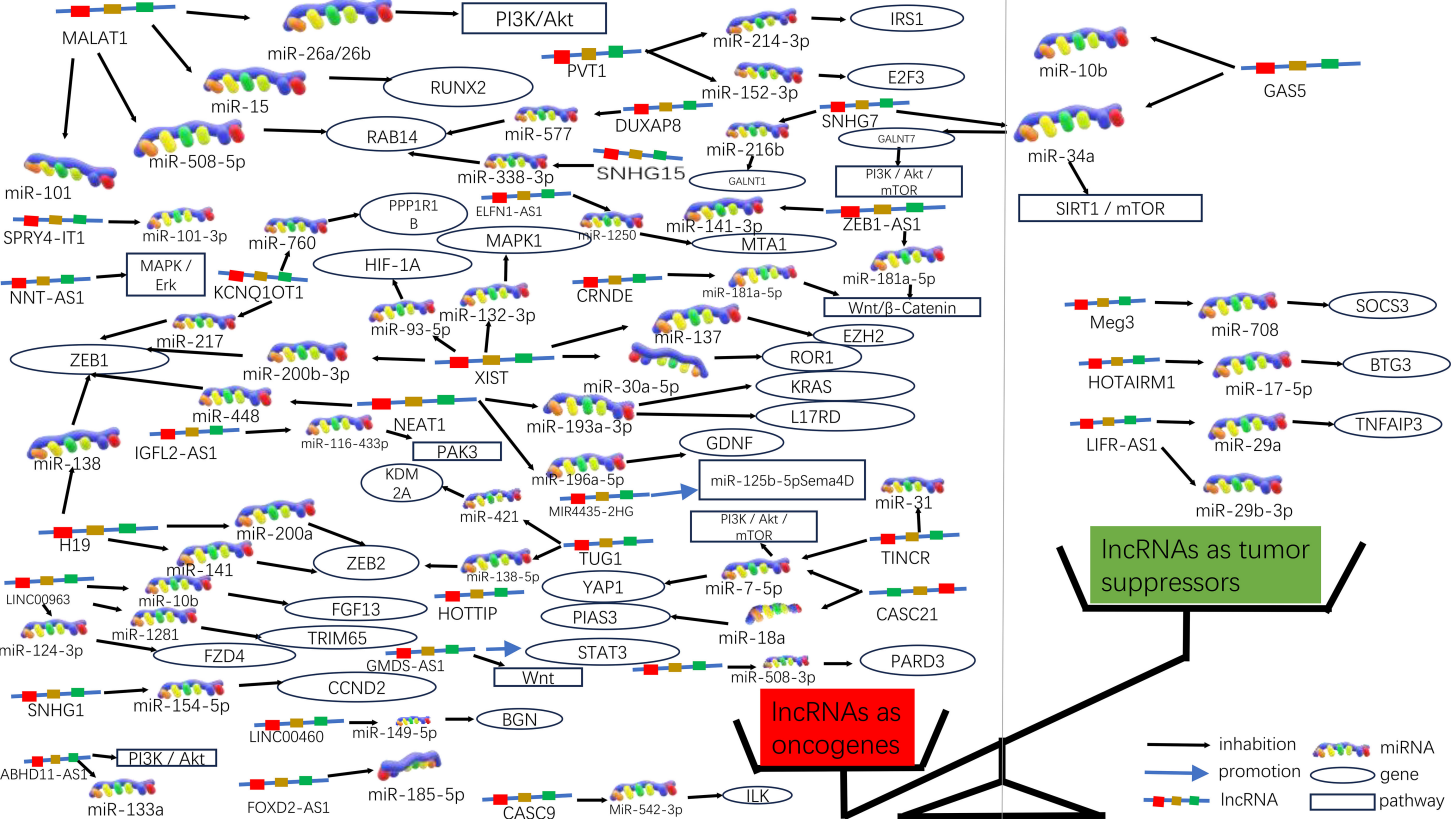
Molecular functions of lncRNAs in CRC progression. Due to the significant differences in lncRNA as oncogenes or oncogenes suppressors, they can be graphically depicted in a scale. On the left side of the scale (red), lncRNAs act as oncogenes in higher numbers, some of which are also involved in signaling pathways; on the right side of the scale (green), lncRNAs act as tumor suppressors in lower numbers.

**Figure 4:**
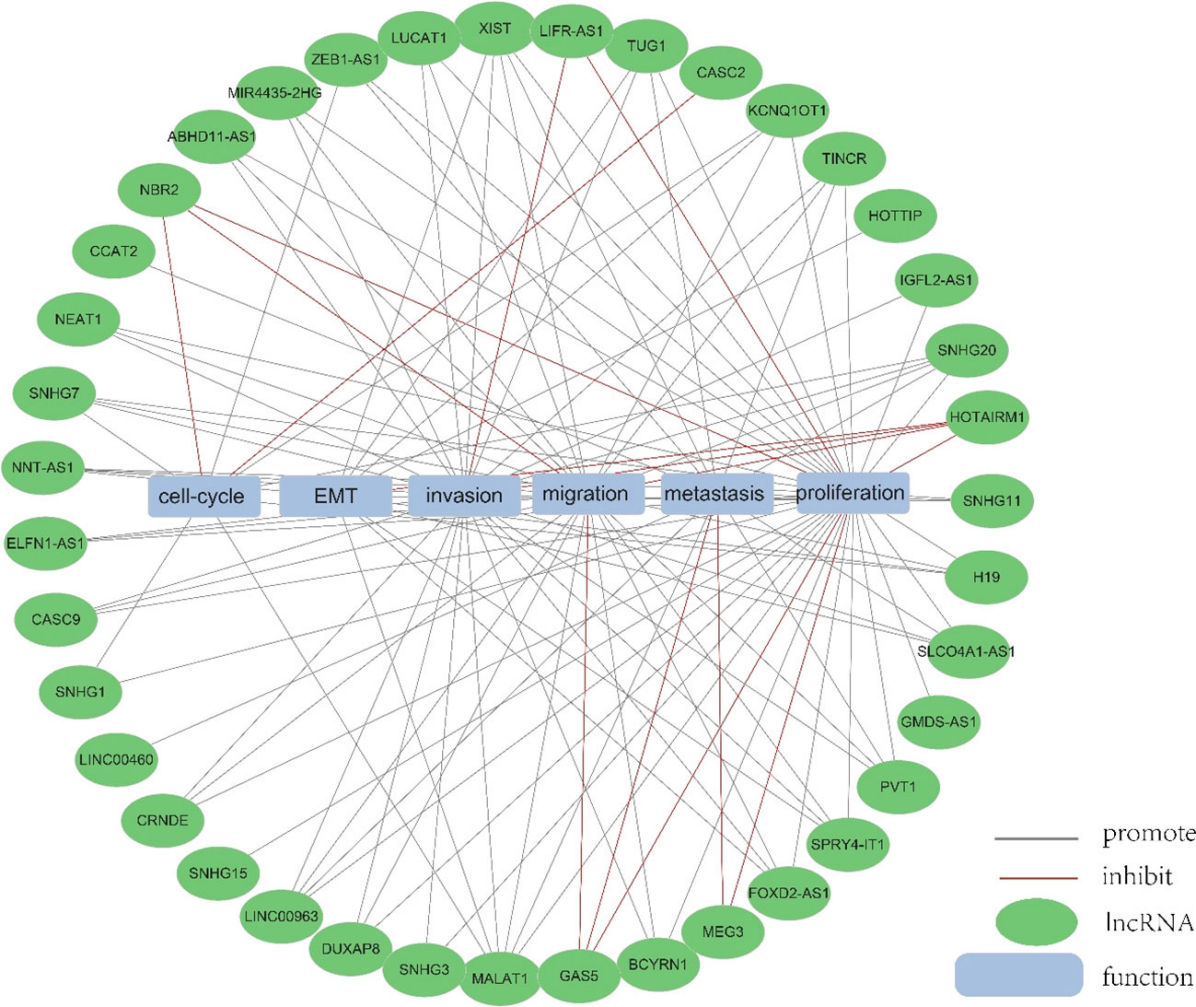
The function of lncRNA. We collected and identified a number of differentially expressed lncRNA functions.

An increasing number of lncRNAs have been identified as playing roles in promoting cancer or inhibiting tumor growth and have significant impacts on the hallmark cancer processes of colorectal cancer initiation, growth and metastasis. **Figures 3** and **4** provide a summary of the aberrantly regulated lncRNAs implicated in these processes in CRC, along with their underlying molecular mechanisms.

#### 3.3.1 LncRNAs as oncogenes in CRC

In 235 publications, 168 aberrantly expressed lncRNAs were reported as oncogenes associated with cell cycle, proliferation, apoptosis, migration, invasion, metastasis, or epithelial-mesenchymal transition in colorectal cancer. Of these, MALAT1, XIST, H19, NEAT1, and PVT1 were investigated in over three studies, with 9, 6, 5, 5, respectively. There are 5 for each lncRNA, and the associated mechanisms are clear and well-established, which will be described in detail in this article.

##### 3.3.1.1 MALAT1

MALAT1 has been identified as a key oncogene, with its increased expression linked to cancer advancement and unfavorable outcomes in CRC (45). In both laboratory and animal studies, it has been demonstrated that MALAT1 is notably increased in CRC and is linked to more advanced TNM staging, lymph node metastasis, and a worse prognosis for patients. MALAT1 promotes the progression of CRC by inhibiting the expression of miR-508-5p, leading to the promotion of RAB14. Knocking down MALAT1 significantly reduces proliferation and invasiveness of colorectal cancer cells in laboratory studies (46). In CRC, the tumor suppressor gene SFPQ can be competitively bound by MALAT1. And PTBP2 be released by MALAT1 from the SFPQ/PTBP2 complex, thereby facilitating tumor growth and migration in colorectal cancer cells (47). MALAT1 could also upregulate FUT4 expression and enhances fucoidan glycosylation and in laboratory studies, MALAT1 triggers the activation of the PI3K/AKT/mTOR pathway, thereby promoting colorectal cancer cell invasiveness (48). Besides, research has found that MALAT1 played a crucial role in promoting the cell cycle progression and EMT (31). Therefore, MALAT1 may serve as a useful therapeutic target in the treatment of CRC. (**Figures 3**, **4**).

##### 3.3.1.2 XIST

XIST expression was significantly elevated in CRC cells, and he significant upregulation of XIST has been found to promote proliferation and migration while inhibiting apoptosis. The upregulation of XIST led to the competitive “sponging” of miR-200b-3p, resulting in the promotion of cell proliferation, invasion, EMT, and stem cell formation *in vitro*. Additionally, this mechanism contributed to tumorigenesis and metastasis *in vivo (* 49). As a competitive sponge for miR-93-5p, XIST may act as a positive regulator of HIF-1A, which in turn increased expression of AXL, and thus promoted the EMT process, as well as migration and proliferation in colorectal cancer (34). Furthermore, lncRNA XIST has been found to facilitate tumor metastasis in colorectal cancer by modulating the miR-137-EZH2 axis (33). Therefore, XIST could potentially be targeted for therapy and used as a prognostic indicator for colorectal cancer. (**Figures 3**, **4**).

##### 3.3.1.3 H19

Increased expression of H19 was correlated with tumor differentiation, TNM staging, and unfavorable prognosis in colorectal cancer. It was found that The H19/miR-194-5p axis regulates the SIRT1-dependent autophagy pathway, which may impact 5-Fu chemoresistance in colon cancer cells. Knockdown of H19 sensitized colon cancer cells to 5-Fu resistance, while enhancing H19 expression improved 5-Fu resistance. These findings suggest that H19 may play a role in the mediation of CRC resistance (50). Additionally, H19 hindered the binding of eIF4A3 protein to mRNAs of cell cycle regulatory genes, resulting in expedited cell cycle progression and increased proliferation of CRC cells (51). In addition to that, H19 acted as a ceRNA for miR-138 and miR-200a, antagonizing their activities and resulting in the release of their endogenous targets Vimentin, ZEB1, and ZEB2 from repression, facilitating the epithelial to mesenchymal transition (52). Thus, H19 could be a promising therapeutic target for the treatment of colorectal cancer (**Figures 3**, **4**).

##### 3.3.1.4 NEAT1

NEAT1 is implicated in the regulation of cell proliferation and migration in colorectal cancer (CRC) through the NEAT1/miR-196a/GDNF pathway. It has been observed that NEAT1 exerts a crucial influence by suppressing miR-196a-5p, leading to an increase in GDNF expression in CRC, consequently facilitating the proliferation and migration of CRC cells (39). NEAT1 exhibited upregulation in both colorectal cancer tissues and cells, and knockdown of NEAT1 inhibited colorectal cancer proliferation and causes decreased ZEB1 expression and increased miR-448 expression. Therefore, NEAT1 could promote ZEB1 expression by targeting and inhibiting miR-448 and promote colorectal cancer proliferation and invasion in this way (38). In addition, NEAT1 increased the level of histone acetylation, and the heightened histone enrichment at the ALDH1 and c-Myc promoters led to the upregulation of ALDH1 and c-Myc expression, thereby impacting 5-Fu resistance in colon cancer cells through modulation of cancer cell stemness (53). Taken together, NEAT1 plays an important role in regulating colorectal malignancy progression. Consequently, NEAT1 can hold promise as a potential therapeutic target for colorectal cancer (**Figures 3**, **4**).

##### 3.3.1.5 PVT1

Studies have shown that the expression of PVT1 is up-regulated in colorectal cancer. and that its upregulated gene expression levels promotes colorectal cancer development. The PVT1 gene functions as a newly discovered oncogenic enhancer of MYC, and its activity is regulated by epigenetic mechanisms involving abnormal methylation in colorectal cancer (54). In colorectal cancer tissues and extracellular vesicles, PVT1 and c-Myc are co-amplified, which in turn promotes the proliferation and metastasis of colorectal cancer cells (55). In addition, The upregulation of PVT1 resulted in elevated mRNA and protein expression levels of multidrug resistance-associated protein 1,and apoptosis regulator Bcl2, which enhanced drug resistance in colorectal cancer cells (56). The PVT1/miR-152-3p/E2F3 axis drives the proliferation, migration, and invasion of colorectal cancer by controlling the transcriptional activation of MAPK8 (57). Studies have shown that silencing PVT1 leads to the suppression of IRS1 and the downregulation of the PI3K/Akt signaling pathway through the upregulation of miR-3-214p. This, in turn, induces apoptosis and restrains the proliferation and invasion of CRC cells (58). Therefore, PVT1 has the potential to serve as a critical diagnostic marker and target for therapeutic intervention.(**Figures 3**, **4**).

#### 3.3.2 LncRNAs exhibit tumor-suppressive roles in CRC

In addition to their roles as oncogenic lncRNAs in CRC, certain lncRNAs act as tumor suppressors by significantly regulating the inhibition of cell proliferation and invasion in CRC. A total of 56 lncRNAs were identified as tumor suppressors across 67 publications. Of these, there are more than 3 studies each on GAS5 and MEG3, respectively, with well-established and comprehensive elucidation of the associated mechanisms, which will be elaborated upon in detail within this paper.

##### 3.3.2.1 GAS5

GAS5 has been found to directly bind to the WW structural domain of YAP, leading to the relocation of YAP from the nucleus to the cytoplasm. This interaction induces the phosphorylation of YAP and triggers its degradation through ubiquitin-mediated pathways., thereby suppressing the progression of colorectal cancer in laboratory settings and in living organisms. In clinical observations, the expression of lncRNA GAS5 showed a negative correlation with the protein levels of YAP and YTHDF3 in the tumors of colorectal cancer patients. It was found that lncRNA GAS5 suppresses the proliferation and migration of colorectal cancer cells and triggers apoptosis through its interaction with miR-10b. It can also serve as a significant regulator of inflammatory cytokines in colorectal cancer occurrence and development via the NF-κB and Erk1/2 pathways, thereby inhibiting tumor cell proliferation (59). In addition, GAS5 is downregulated during CRC progression and functions as a competitive endogenous RNA for miR-34a,which is involved in the regulation of GAS5-inhibited tumor cell autophagy and triggers apoptosis via the mTOR/SIRT1 pathway. GAS5 sustains a homeostatic level of macroautophagy, which may be protective against apoptosis in CRC cells. mTOR signaling pathway suppresses the expression of GAS5 and establishes a feedback mechanism with miR-34a in colorectal cancer cells to mediate CRC cell macroautophagy (60). Therefore, GAS5 may serve a crucial role as an important tumor suppressor in tumor therapy (**Figures 3**, **4**).

##### 3.3.2.2 MEG3

Studies have shown that MEG3 overexpression suppresses the proliferation and metastasis of colorectal cancer cells, but in colorectal cancer tissues and cells, there is a significant downregulation of MEG3 expression. Additionally, miR-708 enhances STAT3 activation by targeting the 3’UTR of SOCS3, and the decreased expression of MEG3 in colorectal cancer (CRC) tissues and cells leads to a reduction in SOCS3 levels, promoting the malignant proliferation of colon stem cells and CRC cell lines. MEG3 acts as a sponge for miR-708, inhibiting the malignant proliferation of colon stem cells in colorectal cancer (61). MEG3 exhibits downregulation in colorectal cancer and exerts dual effects on cell behavior. On one hand, it promotes cell proliferation, while on the other hand, it induces apoptosis in colorectal cancer. This is achieved by targeting adenosine deaminase to RNA 1 in CRC (62). In addition, The vitamin D receptor (VDR) directly binds to the promoter of MEG3, leading to the stimulation of its expression in CRC cells. This upregulation of MEG3 exerts tumor suppressor effects in colorectal cancer by regulating the activity of Clusterin. This mechanism may explain the anticancer activity of vitamin D on CRC cells (63). Therefore, MEG3 holds promise as a viable therapeutic target and diagnostic biomarker for colorectal cancer treatment (**Figures 3**, **4**).

### 3.4 LncRNA as a biomarker

#### 3.4.1 LncRNA as a diagnostic marker for CRC

Among the screened literature, five lncRNAs including XIST, SLC7A11-AS1, NEAT1, MALAT1 and CCAT2, PVT1, and SNHG11 have been reported to be up-regulated in CRC in several papers, MALAT1 (48, 64–66) and NEAT1 (39, 67–69) were reported to be overexpressed in four papers, respectively.MALAT1 showed higher levels in tumor tissues and was associated with metastasis in CRC patients (48) PVT1, SNHG11 is significantly upregulated in CRC patients (64, 70) PVT1, SNHG11.

In addition, there are 2 papers each showing that XIST (34, 71) and CCAT2 (72, 73) were significantly upregulated. On the other hand, HOTAIRM1 (74) was reported to be significantly downregulated in two publications. These lncRNAs did not report inconsistent results in 78 publications. Thus these significantly up- and down-regulated lncRNAs have the potential to be developed as diagnostic markers.

#### 3.4.2 LncRNA as a prognostic marker for CRC

In 131 papers, it was proposed that lncRNAs could be used as prognostic markers for CRC. Among them, LUNAR1, SNHG3, LUCAT1, PVT1, MALAT1, XIST, MEG3, and NEAT1 were significantly changed in more than 2 studies. Elevated expression of XIST, PVT1, SNHG3, MALAT1, and NEAT1 were all related with poor prognosis.

LncRNA XIST (33, 75) Overexpression predicts poor PFS and poor OS in CRC patients.PVT1 is the most frequently studied lncRNA as a prognostic biomarker, and 2 publications have shown that upregulation of PVT1 is associated with poorer overall survival (OS) and disease-free survival (DFS) (54, 76). In 4 studies, MALAT1 (47) was an important prognostic biomarker (45). MALAT1 had a significantly higher expression in CRC with poor prognosis. High NEAT1 expression is an independent prognostic marker of poor outcome and an important indicator of tumor recurrence in colorectal cancer (37). In addition, lncRNA MEG3 (62) is downregulated in CRC and can be used as a prognostic marker. SNHG3 acts as a competitive endogenous RNA (ceRNA) that “sponges” miR-182-5p, leading to the release of c-Myc from miR-182-5p and regulating c-Myc expression (77). Therefore, overexpression of SNHG3 is thought to be associated with poor prognosis of CRC. Studies have shown that LUNAR1 inhibition significantly suppresses IGF1 signaling in CRC, and LUNAR1 may be a promising prognostic marker for clinical management of colorectal cancer (78). High lncRNA BANCR expression is associated with poor OS and linked to lymph node metastasis and poor survival in colorectal cancer. LncRNA BANCR may serve as a novel and useful biomarker for CRC lymph node metastasis and prognosis (79, 80). Two studies suggested that LUCAT1 was upregulated in CRC tissues; and colorectal cancer patients with higher LUCAT1 had a poorer clinical prognosis (81, 82).

#### 3.4.3 LncRNA as a biomarker of therapeutic response

Thirty-seven studies in the literature focused on lncRNAs as biomarkers for predicting response to CRC therapy, and in these papers, the results suggested that MEG3, CACS15, GAS5, and LUCAT1, which are lncRNAs, are biomarkers for drug therapy.

MEG3 acts as a ceRNA for miR-141, which leads to miR-141 inhibition (83) which in turn de-represses the downstream target PDCD4. notably, PDCD4 sensitizes cancer cells to cisplatin, paclitaxel, and doxorubicin by acting as a tumor suppressor to induce apoptosis. This study demonstrates that overexpression of MEG3 may serve as a potential strategy for therapeutic outcomes in CRC. CASC15 is upregulated in oxaliplatin (OXA)-resistant CRC cells (84), while CASC15-silenced OXA-resistant CRC cells restored sensitivity to OXA. Mechanistically, CACS15 promotes oxaliplatin resistance in CRC cells by elevating ABCC145 through the CACS1/miR-15 axis. In this context, downregulation of CASC15 may be a therapeutic strategy to promote response to oxaliplatin in CRC patients. GAS5 can inhibit CRC progression *in vitro* and *in vivo* by directly interacting with the WW structural domain of YAP, facilitating translocation of endogenous YAP from the nucleus to the cytoplasm, and promoting phosphorylation and subsequent ubiquitin-mediated degradation of YAP (85). LUCAT1 binds to UBA40 encoding ubiquitin and 52S ribosomal protein L60 (RPL40). We found that RPL40 acts in the ribosomal protein-MDM2-p53 pathway to regulate p53 expression (82). Knockdown of LUCAT1 sensitizes CRC cells to oxaliplatin treatment and may be able to make LUAT1 a novel therapeutic target.

### 3.5 Screening and validation of Hub genes

#### 3.5.1 Construction of the PPI network

**Figure 5:**
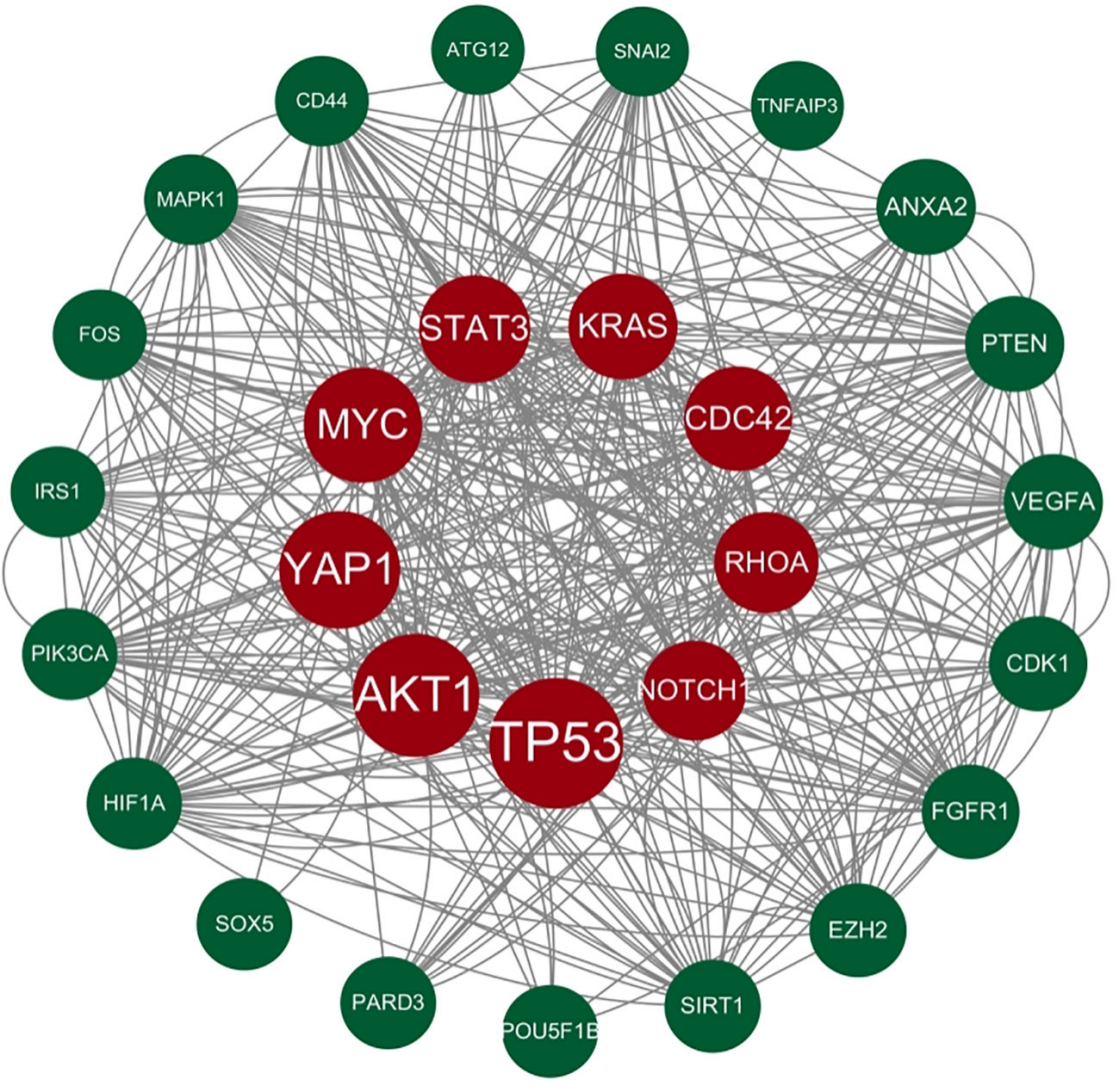
Hub gene gained from CytoNCA. Hub genes were calculated in CytoNCA using Betweenness Centrality and ranked according to their score scores (Appendix 1), the top 28 scores are shown in this figure. The genes in the middle red are usually considered to be the highest scoring and the larger they are the higher the score proved to be.

**Figure 6:**
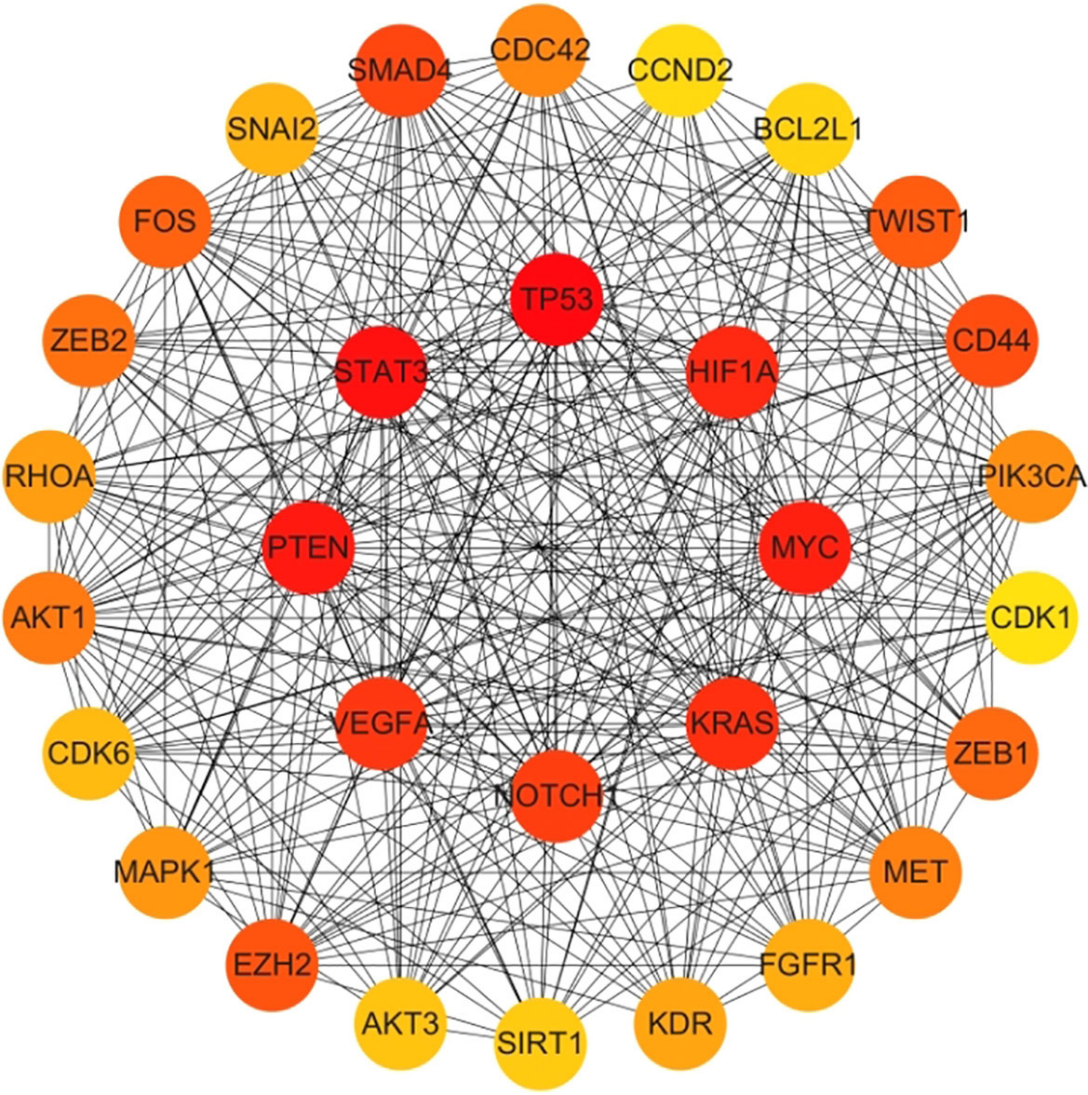
Hub gene gained from MCC. Hub genes were calculated in Cytohubba using MCC, and ranked in order of their relevance (Appendix 2), with the top 30 scores as shown in this figure. These genes in the inner circle are considered to be the genes with higher scores as calculated by MCC, and the outer circle has lower scores in comparison.

**Figure 7:**
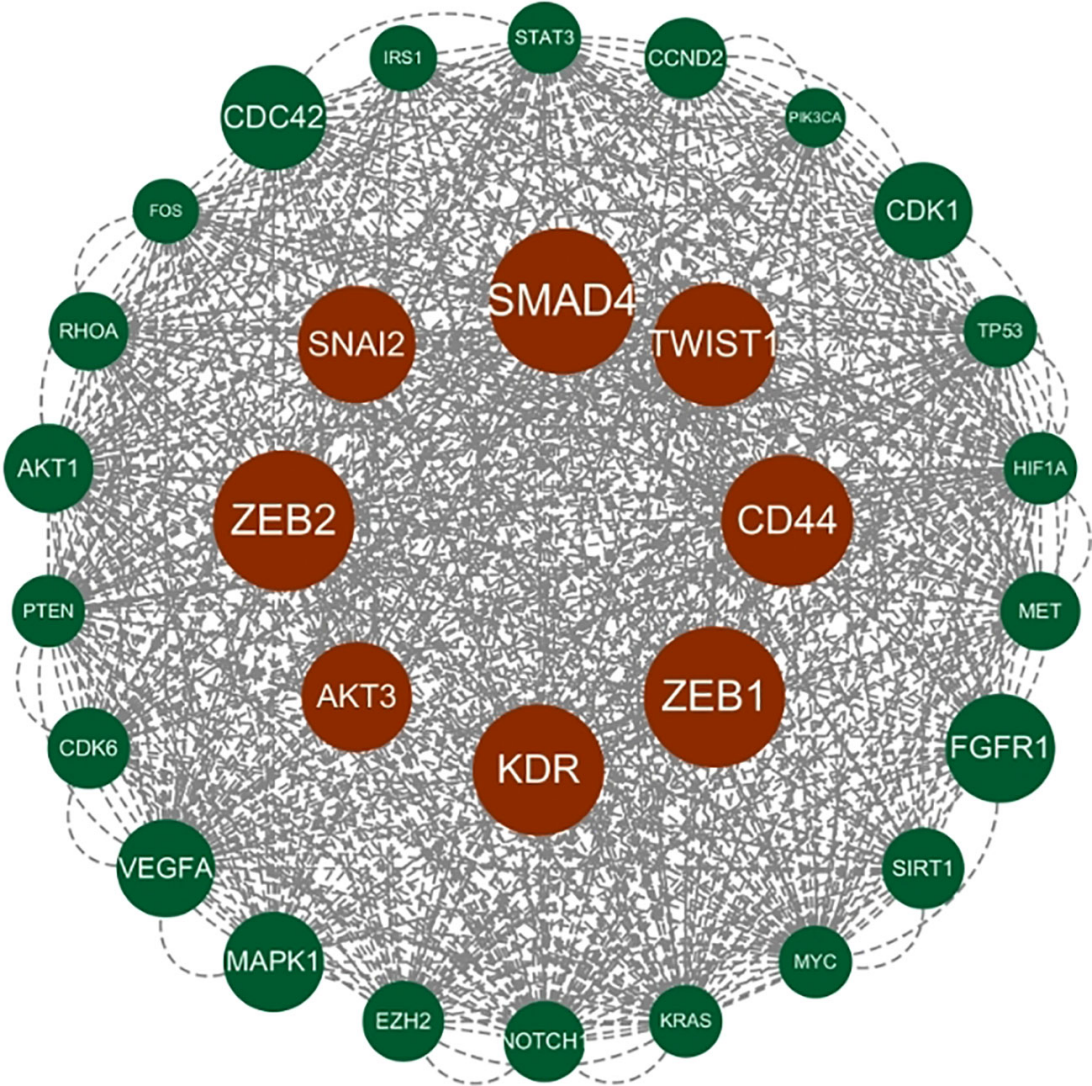
Hub gene gained from MCODE. Hub genes were derived from MCODE calculations (Appendix 3) and ranked based on the size of the scores, with the top 30 scores as shown in this figure. These genes in the inner circle are considered to be the genes with higher scores as calculated by MCODE, and the outer circle has lower scores in comparison.

**Figure 8:**
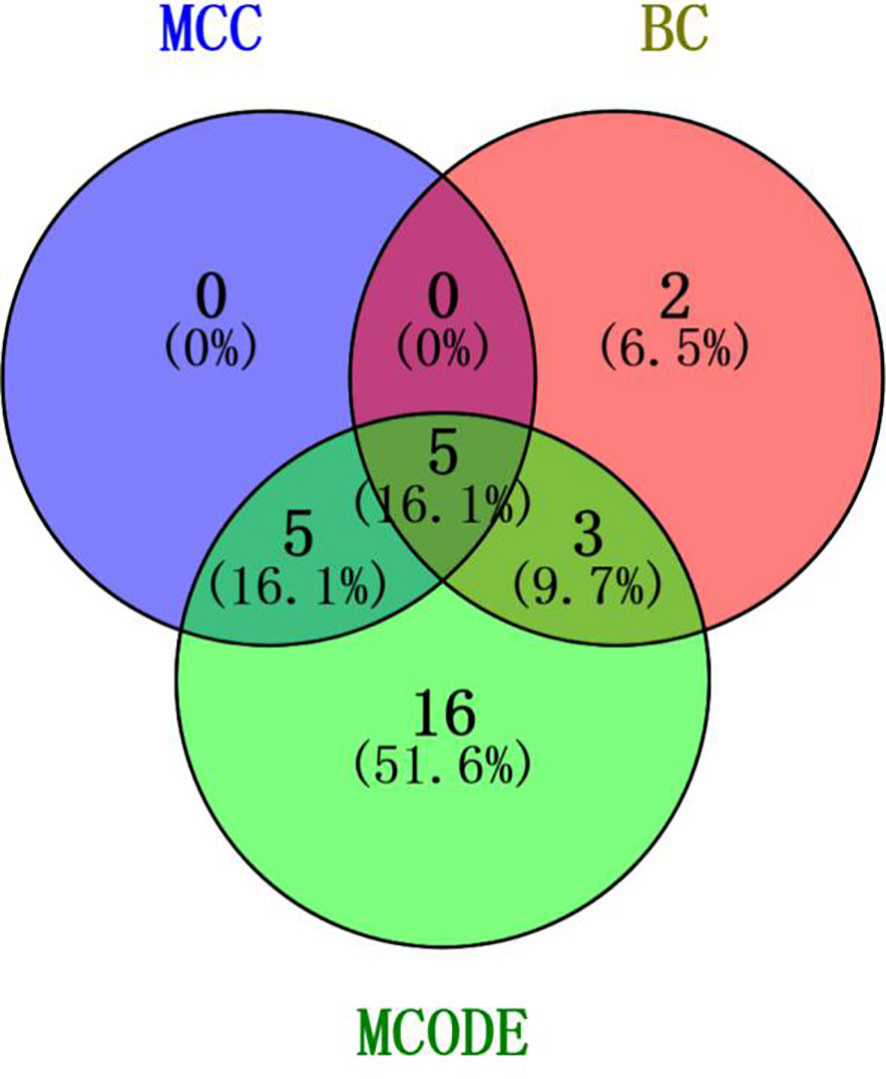
Intersection of core genes. Taking the top ten with high MCC scores, the top ten with high BC scores, and the top 29 validated by MCODE to obtain the intersection, which in turn identified our five hub genes as TP53, MYC, STAT3, KRAS, and NOTCH1.

The PPI network was constructed by inputting lncRNA-related genes into STRING, and the results were imported into the Cytoscape (3.10.0) software for further screening of hub genes, with the following results:

Genes involved in lncRNA were screened using 360 known publications and imported into the string to perform protein interaction analysis. Immediately following this, hub genes were calculated using Betweenness Centrality in CytoNCA using Cytoscape (3.10.0) and ranked according to their score scores (Appendix 1), the top 28 scores are shown in **Figure 5**. Hub genes were calculated using MCC in Cytohubba and ranked according to their relevance (Appendix 2), and the top 30 scores are shown in **Figure 6**. Hub genes derived from MCODE calculations (Appendix 3) and sorted according to the size of their scores, and the top 30 scores are shown in **Figure 7**. The top ten with high MCC and BC scores and the top 29 validated by MCODE were taken to obtain the intersection, which in turn identified our five hub genes as TP53, MYC, STAT3, KRAS, and NOTCH1 (**Figure 8**).

#### 3.5.2 Differential expression analysis of hub genes

**Figure 9:**
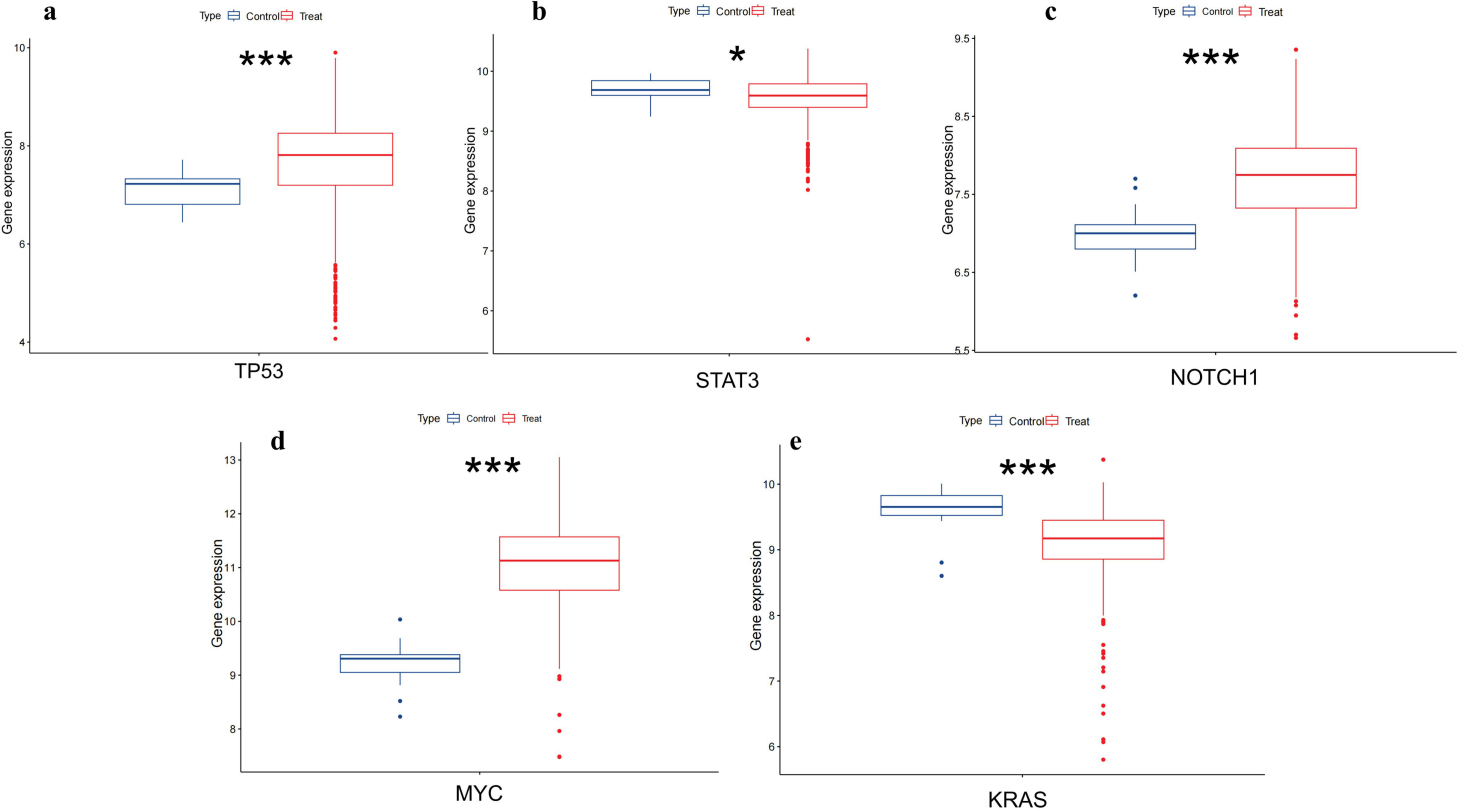
Differential expression of TP53,MYC,STAT3,NOTCH1 and KRAS in colorectal tissues. **(A)** shows that TP53 is upregulated in colorectal cancer(*p<0.001*). **(B)** shows that STAT3 is low expressed in colorectal cancer(*p<0.05*). **(C)** shows NOTCH1 is upregulated in colorectal cancer(*p<0.001*). **(D)** shows that MYC has upregulation in colorectal cancer(*p<0.001*). **(E)** shows that KRAS has downregulation in colorectal cancer(*p<0.001*). (*, p<0.05；**, p<0.01; ***, p<0.001).

To further validate the differential expression of the two genes in colorectal cancer, we utilized the pan-cancer dataset from the GEO database:GSE39582, from which further we extracted TP53,MYC,STAT3,KRAS and NOTCH1 genes in colorectal cancer samples, and calculated the expression differences between normal and tumor samples using R software (version 4.4.1), and the expression differences between normal and tumor samples in each tumor using unpaired Wilcoxon Rank Sum and Signed Rank Tests for significance of difference analysis, it was observed that TP53,MYC and NOTCH1 were significantly up-regulated in colorectal cancer. STAT3 and KRAS were significantly down-regulated in colorectal cancer (**Figure 9**).

#### 3.5.3 Validation of GEPIA gene expression

**Figure 10:**
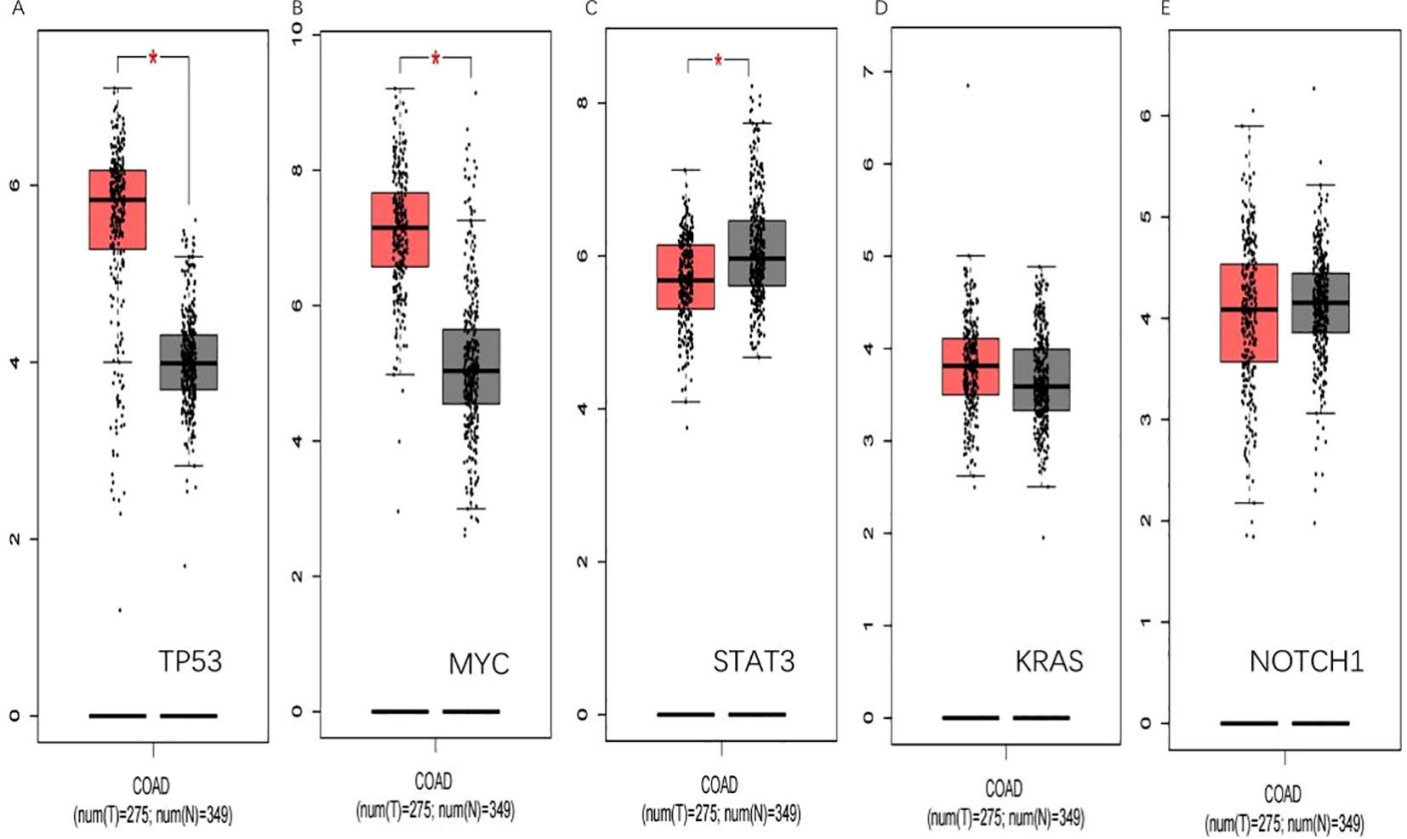
GEPIA verification. **(A)** shows that TP53 is significantly upregulated in colorectal cancer(*p<0.05*).**(B)** shows that MYC is significantly upregulated in colorectal cancer(*p<0.05*). **(C)** shows STAT3 is low expressed in colorectal cancer(*p<0.05*). **(D)** and **(E)** show that KRAS and NOTCH1 have been upregulated in colorectal cancer. (*, p<0.05).

In the GEPIA database, the expression of the five hub genes screened was verified with the following results (**Figure 10**).

In the previous account, lncRNAs are involved in colorectal cancer development as pseudogenes; the lncRNA-protein axis is involved in colorectal cancer development, and lncRNAs can be used as structural components to regulate protein activity or alter protein localization by binding to specific proteins containing nuclear transcription factors; and the lncRNA-miRNA-mRNA axis regulates CRC gene expression (22).Through these regulatory processes, lncRNAs are linked to gene expression. The screened hub genes were shown to be significantly associated with the development of colorectal cancer.

Through the validation of differential gene expression in the GEPIA database, we found that the validation of MYC and STAT3 in the GEPIA database yielded the same results as the GEO database, which could be able to serve as targets to play a role as tumor marker in colorectal cancer.

#### 3.5.4 Expression of hub genes

**Figure 11:**
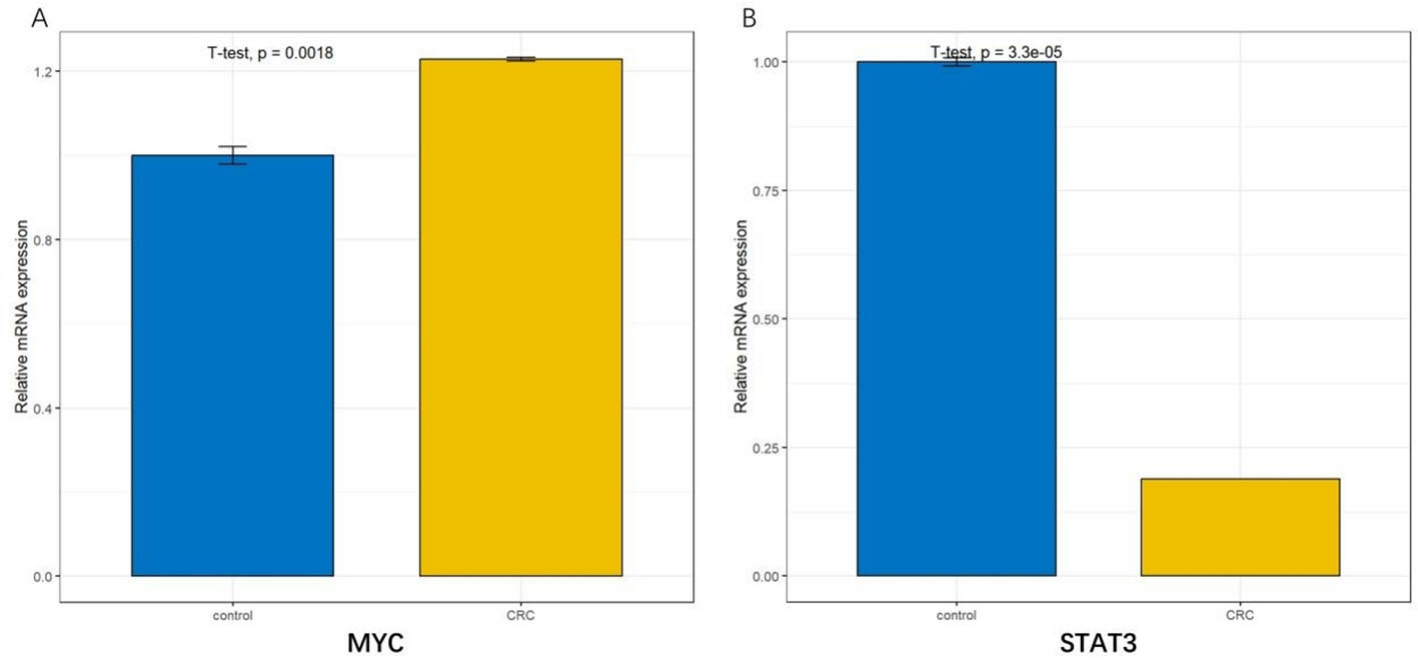
qRT-PCR results. The difference in the expression of Hub genes in normal colorectal and colorectal cancer cells. **(A)** showed that the MYC was upregulated in colorectal cancer (*p=0.0018*); **(B)** showed that STAT3 was downregulated in colorectal cancer cells (*p=3.3e-05*).

The scope of the qRT-PCR technique makes it suitable for a wide range of experimental conditions and allows experimental comparisons between normal and cancer tissues. β-actin was selected as the internal reference gene for independent validation. The qRT-PCR experimental results demonstrated that the MYC gene was upregulated(*p=0.0018*), while the STAT3 gene was downregulated(*p=3.3e-05*) in colorectal tissue (**Figure 11**).The experimental results were consistent with those validated in two databases. So the screened hub genes are expected to be novel colorectal cancer marker genes.

#### 3.5.5 Determination of ROC curves

**Figure 12:**
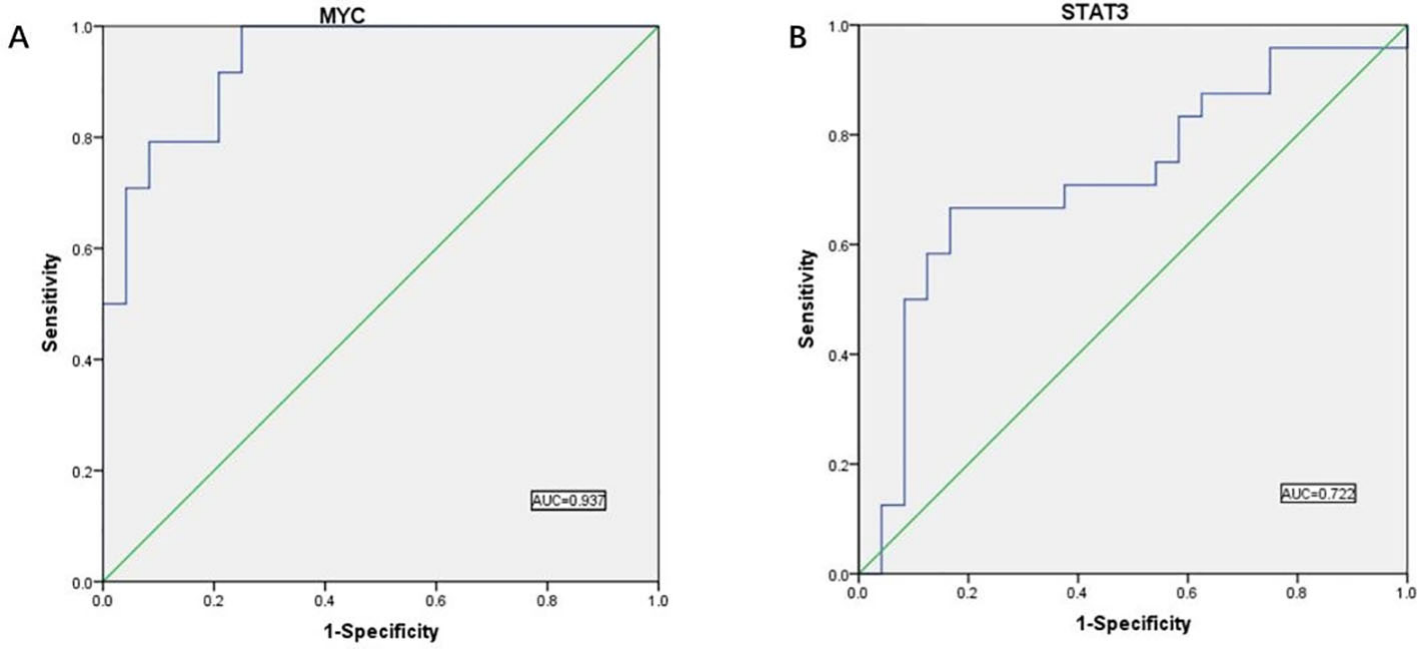
ROC curve results. **(A)** ROC curve analysis showed the predictive performance of the MYC model. The AUC value was 0.937 (95% CI: 0.874-1.000), indicating that the model has good predictive accuracy. **(B)** ROC curve analysis showed the predictive performance of the STAT3 model. the AUC value was 0.722 (95% CI: 0.572-0.872), indicating that the model has good predictive accuracy.

To further test its validity as a diagnostic biomarker, we relied on the dataset of GSE10950 from the GEO database to perform ROC analysis on the hub genes we have screened, and the images obtained after SPSS processing are shown in **Figure 12**.

AUC values above 0.7 indicate that the gene has significant diagnostic value for use in the diagnostic process of CRC disease. We found validated two hub genes with AUC values of 0.937 and 0.722, respectively, that were shown to be useful as diagnostic biomarkers in colorectal cancer. It is reasonable to assume that MYC and STAT3 have significant diagnostic value.

#### 3.5.6 Immunohistochemical results of STAT3 and MYC

**Figure 13:**
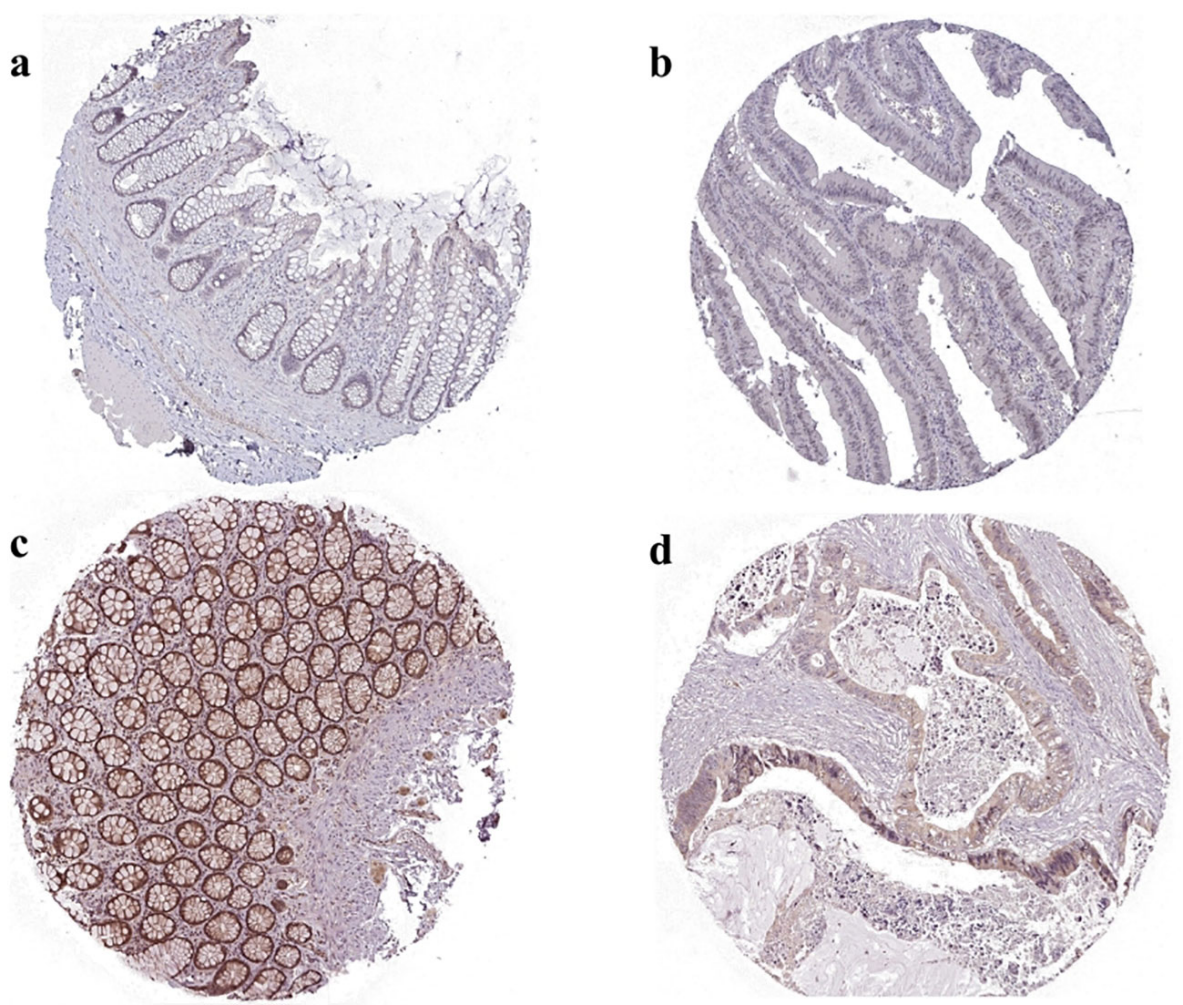
Immunohistochemical results of MYC and STAT3 in normal and tumor tissues in HPA databases. **(A)** MYC expression in normal tissues. The normal tissue source chosen was normal rectal tissue from a 58 year old woman with antibody HPA055893, with a low degree of immunohistochemical staining and intensity. **(B)** MYC expression in cancerous tissues. The cancerous tissue source chosen was cancer rectal tissue from a 81 year old woman with antibody HPA058603, with a high degree of immunohistochemical staining and intensity. **(C)** STAT3 expression in normal tissues. The normal tissue source chosen was normal rectal tissue from a 62 year old woman with antibody HPA058603, with a high degree of immunohistochemical staining and intensity. **(D)** STAT3 expression in cancerous tissues. The cancerous tissue source chosen was cancer rectal tissue from a 63 year old man with antibody HPA058603, with a low degree of immunohistochemical staining and intensity.

In order to further validate the differential expression of the two genes in colorectal cancer, we selected specific antibodies HPA058603 against STAT3 and HPA055893 against MYC for staining by mining the HPA database and mapped the two sets of immunohistochemistry results in order to assess the differences in the expression patterns of these genes in colorectal cancer. We observed significant differences in the expression levels of STAT3 and MYC in colorectal cancer tissues. Colorectal tissues with upregulation of MYC and downregulation of STAT3 were significantly different from normal colorectal tissues (**Figure 13**).

#### 3.5.7 Function of the hub genes

MYC transcription factors are key regulators of cell growth and proliferation and are established targets for cancer therapy. c-MYC, a pleiotropic transcriptional regulator, integrates essential processes required for normal cell proliferation and survival (86–88). c-MYC functions as both a transcriptional activator and repressor, coordinating the expression of a large and diverse set of genes. These genes regulate intracellular functions (such as cell growth, cell cycle progression, biosynthetic metabolism, and apoptosis) as well as extracellular processes that synchronize cell proliferation with the surrounding somatic cellular microenvironment (such as angiogenesis, invasion, matrix remodeling, and inflammation) (89, 90). c-MYC has also been identified as immunosuppressive (91). Deregulated expression of c-MYC protein is observed in a wide range of human cancers and is particularly associated with aggressive disease and poor clinical outcomes (92). In colorectal cancer, lncRNA PVT1 has been reported to stabilize MYC protein (93). Additionally, the PVT1 locus may function as an enhancer directly controlling MYC expression. Targeting PVT1 could be a potential therapeutic approach for colorectal cancer patients, potentially leading to the inhibition of oncogenic MYC at both RNA and protein levels (54). NCAPD3 upregulates c-Myc levels and interacts with c-Myc, recruiting it to downstream glycolytic regulators such as GLUT1, HK2, ENO1, PKM2, and LDHA gene promoters, ultimately enhancing cellular aerobic glycolysis, promoting the Warburg effect, and driving colorectal cancer development and progression (94). c-Myc also upregulates the expression of lncRNA SNHG15, which promotes colorectal cancer development and mediates drug resistance (95).

In CRC, STAT3 affects T cell activity by inhibiting the function of antigen-presenting cells, and when STAT3 expression is inhibited, the tumor cells’ ability to evade the immune system is weakened, allowing the immune system to more effectively recognize and eliminate cancer cells (96). STAT3 is activated by interleukin-6 (IL-6), promoting tumorigenesis and progression (97). IL-6/STAT3 signaling plays a crucial role in modulating tumor-infiltrating immune cells within the tumor microenvironment. This pathway activates downstream genes that protect tumor cells from apoptosis, drive cell proliferation, cell cycle progression, invasion, metastasis, stimulate angiogenesis, and induce drug resistance (98). In CRC cells, GMDS-AS1-HuR interactions protect HuR from degradation, stabilizing STAT3 mRNA, upregulating basal and phosphorylated STAT3 protein levels, and maintaining continuous STAT3 pathway activation. Studies have shown that GMDS-AS1 and its direct target, HuR, collaboratively activate the STAT3/Wnt signaling pathway, contributing to CRC progression. The GMDS-AS1-HuR-STAT3/Wnt axis represents a potential therapeutic target for the treatment, diagnosis, and prognosis of colorectal cancer (99). In the early stage of colorectal cancer, tumor cells are highly differentiated and relatively less malignant, and their STAT3 levels are low expressed. In addition, a family of proteins controlling STAT protein factors has been identified, including protein-activated STATs inhibitors (PIAS) (100). Many components of this protein family have been identified (PIAS1, PIAS3, PIAS5, PIASy), which regulate the transcriptional activity of many transcription factors through different mechanisms. For example, PIAS3 binds to dimeric STAT3 and inhibits its DNA-binding ability, thereby interfering with STAT3-mediated gene activation resulting in decreased STAT3 expression levels. STAT3 is silenced in colorectal cancer cells due to epigenetic modifications (methylation), resulting in decreased expression (101).

## 4 Discussion

This study establishes the potential value of MYC and STAT3 as biomarkers for CRC, providing new diagnostic and therapeutic targets. It was found that MYC was upregulated in CRC, while STAT3 was downregulated in CRC, and these two genes had significant diagnostic value by ROC curve analysis. However, the study in this paper has some limitations, such as failing to comprehensively cover the changes in lncRNA expression in different clinical sample types. In addition, future studies should aim to further validate the applicability of MYC and STAT3 as CRC markers and delve into their interactions with lncRNAs to better support their application in early screening and personalized treatment of CRC.

The advancement of high-throughput sequencing technology and bioinformatics tools has revealed that the abnormal expression of lncRNAs is linked to the initiation and advancement of various cancers, such as CRC (102). These lncRNAs play a role in the biological processes of different cancers, impacting cell growth, programmed cell death, movement, and the spread of cancer cells (103). Earlier evaluations and analyses have highlighted the predictive significance of lncRNAs in numerous cancer types, including colorectal cancer, ovarian cancer (104), lung cancer (105), etc. Nevertheless, studies on CRC are still insufficient. Hence, this article was conducted to underscore the significance of lncRNAs in CRC. We performed a detailed summary of 360 screened publications on colorectal cancer and lncRNAs to provide an overview for the role of lncRNAs in cancer. It was summarized that some lncRNAs have oncogenic effects, such as MALAT1 and XIST (106, 107), which trigger the development of colorectal cancer; and the other part of lncRNAs have oncogenic effects, such as GAS5 and MEG3 (63, 85), inhibitory effects on cell proliferation and invasion in CRC. Meanwhile there is also a part of lncRNAs that can be used as biomarkers for diagnosis, such as SNHG3 and LUNAR1 (78, 108). The role of these lncRNAs reveals the important potential of lncRNAs in colorectal cancer development.

LncRNAs have been shown to be important in the diagnosis and treatment of colorectal cancer. And lncRNAs mainly act through the ceRNA network (109). We innovatively used lncRNAs for PPI network construction to screen differential genes and validated the screened genes in various aspects. Through extensive literature search, we performed a pivotal gene screen using Cytoscape and finally identified five pivotal genes. The are TP53, MYC, STAT3, KRAS, and NOTCH1. Immediately afterward, we used the GSE39582 dataset in geo database and the GEPIA database for screening, and obtained two genes MYC and STAT3 whose expression levels were consistent in the two databases. Then we used qRT-PCR to verify the two genes at the RNA level, and the results were consistent with the previous database verification. We then used the results of immunohistochemistry in the HPA for further validation. Finally, we performed ROC curve mapping using the GSE10950 dataset in the GEO database, demonstrating the potential of these two genes as prognostic markers.

The hub genes, MYC and STAT3, screened by lncRNA corroborate the literature for both genes. The study confirms their important role in increased cancer incidence, cell proliferation and metastasis. These findings provide important targets for future clinical treatment and provide a new perspective and theoretical basis for early diagnosis and treatment of colorectal cancer. The article starts from the ceRNA pathway to screen the hub genes, and screens the genes through the ceRNA regulatory network, and also reveals the potential connection between these two CRC biomarkers and lncRNAs, which provides new insights and potential clues for the mechanistic study of colorectal cancer. The clinical applicability of MYC and STAT3 in CRC remains a challenge, particularly due to variability in expression among different patients, as well as their sensitivity and specificity in early screening. Furthermore, their role in personalized therapy requires deeper investigation, especially in developing targeted treatment strategies tailored to individual patients. Looking ahead, MYC and STAT3 may serve as promising therapeutic targets for CRC. Integrating research on the regulatory mechanisms of lncRNAs could pave the way for novel therapeutic approaches, enhancing the clinical translational potential of CRC treatment. For instance, inhibiting the interaction between lncRNAs and MYC or STAT3 could suppress proliferation and migration in CRC cells, thereby offering new therapeutic avenues.

## Data Availability

The original contributions presented in the study are included in the article/supplementary files. Further inquiries can be directed to the corresponding authors.
